# Identification of apple cultivars hypoallergenic for birch pollen‐allergic individuals by a multidisciplinary in vitro and in vivo approach

**DOI:** 10.1002/clt2.12186

**Published:** 2022-08-26

**Authors:** Maria R. Strobl, Ute Vollmann, Julia Eckl‐Dorna, Astrid Radakovics, Verena Ibl, Madeleine Schnurer, Martin Brenner, Georgi Dermendjiev, Wolfram Weckwerth, Michael Neumüller, Florian Frommlet, Hilal Demir, Merima Bublin, Christian Müller, Barbara Bohle

**Affiliations:** ^1^ Institute of Pathophysiology and Allergy Research, Center for Pathophysiology, Infectiology and Immunology, Medical University of Vienna Wien Austria; ^2^ Department of Otorhinolaryngology Medical University of Vienna Vienna Austria; ^3^ Department of Functional and Evolutionary Ecology Division of Molecular Systems Biology Faculty of Life Sciences University of Vienna Vienna Austria; ^4^ Bavarian Centre of Pomology and Fruit Breeding Hallbergmoos Germany; ^5^ Center for Medical Statistics, Informatics and Intelligent Systems, Section for Medical Statistics, Medical University of Vienna Vienna Austria

**Keywords:** apple, basophil activation test, birch pollen‐associated food allergy, Mal d 1

## Abstract

**Background:**

Birch pollen‐related apple allergy is the most frequent IgE‐mediated food allergy in Central‐Northern Europe with Mal d 1 as major allergen. Its concentration in apples varies with the cultivar and storage time. Year‐round appealing, hypoallergenic cultivars still are needed to satisfy the nutritional needs of affected individuals. We characterized three promising cultivars by multidisciplinary in vitro assays including long‐term storage and by clinical challenges of allergic individuals before and after the birch pollen season.

**Methods:**

Proteins were extracted from fruits of ‘Santana’, ‘Golden Delicious’ (GD), and three genuine cultivars in November 2018 and April 2019. Mal d 1‐levels were analysed by mass spectrometry, SDS‐PAGE, immunoblotting, competitive ELISA, and basophil activation tests. Twenty‐eight allergic individuals underwent single‐blinded open food challenges and skin testing with the cultivars and birch pollen in November 2018 and May 2019. Allergen‐specific IgE‐levels were determined.

**Results:**

After storage all cultivars except ‘Santana’ were of appealing appearance and taste. Their Mal d 1 content had increased, also reflected by significantly amplified basophil activation and stronger reactions in clinical challenges. Besides, individuals showed boosted reactivity after pollen exposure indicated by enhanced allergen‐specific IgE‐levels and skin reactions to birch pollen. Still, all cultivars remained significantly less allergenic than GD and comparable to Santana in November 2018 in all assessments except for skin testing.

**Conclusions:**

Combined expertise in pomology and allergology identified promising new cultivars for allergic consumers. The evaluation of hypoallergenic apples should incorporate long‐term storage and birch pollen exposure. Basophil activation tests may be suitable in the selection of promising cultivars for oral challenges.

**Abbreviations**
BAT: basophil activation test
BIP2: binding immunoglobulin protein
BPRAA: birch pollen‐related apple allergy
BSA: bovine serum albumin
DTT: dithiothreitol
GD: Golden Delicious
OAS: oral allergy syndrome
o.n.: overnight
RT: room temperature
PtP: prick‐to‐prick
VAS: visual analogue score

## 1 INTRODUCTION

Birch pollen‐related food allergy is the most common IgE‐mediated food allergy in Central and Northern Europe and often caused by apple fruits (*Malus domestica*).^1^, ^2^, ^3^, ^4^ Birch pollen‐related apple allergy (BPRAA) is predominantly caused by the major apple allergen, Mal d 1, which is structurally related to the major birch pollen allergen, Bet v 1.^2^, ^5^, ^6^, ^7^, ^8^ Following respiratory sensitization, a fraction of Bet v 1‐specific IgE antibodies cross‐reacts with Mal d 1 and may cause immediate reactions to fresh apples. Most commonly this cross‐reactivity manifests as the so‐called oral allergy syndrome (OAS) which is characterized by itching, tingling, and swelling in the mouth or oral angioedema.^9^ Moreover, Mal d 1 may activate Bet v 1‐specific effector T cells and trigger allergic late phase reactions which appear as an aggravation of atopic eczema.^4^, ^10^, ^11^, ^12^, ^13^ In many individuals BPRAA persists perennially and worsens during or shortly after the birch pollen season.^2^, ^14^ Consequently, around 9% of individuals living in Central Europe refrain from consuming fresh apples.^15^ However, these fruits represent an important domestic source of vitamins, secondary plant metabolites such as phenolic compounds, carbohydrates, and fibers in the diet of the population in areas where birch trees are abundant. Therefore, “hypoallergenic” apple cultivars tolerable by birch pollen‐allergic individuals will help to comply with their nutritional needs.

In the past, numerous studies have compared the allergenic potential of different cultivars by oral challenges and/or skin testing of individuals with BPRAA.^16^, ^17^, ^18^, ^19^, ^20^, ^21^, ^22^, ^23^, ^24^ Concordantly, ‘Golden Delicious’ (GD) was defined as a cultivar of high and ‘Santana’ as a cultivar of low allergenic potential.^17^, ^25^, ^26^, ^27^ Due to positive agronomic and economic traits, good taste, and year‐round availability, GD is the most popular *Malus domestica* cultivar in Europe for decades. In contrast, the fruits of Santana cannot be stored longer than 3 months after harvest and become soft and inedible thereafter. Even modern storage conditions, such as controlled atmosphere storage and treatment with 1‐methylcyclopropene (1‐MCP), hardly overcome this disadvantage. Several other hypoallergenic apple varieties described so far also have significant disadvantages regarding cultivation and storability. Consequently, the number of hypoallergenic cultivars with good agronomic performance (also for organic farming), excellent storability and quality of the fruits is still small.

**FIGURE 1:**
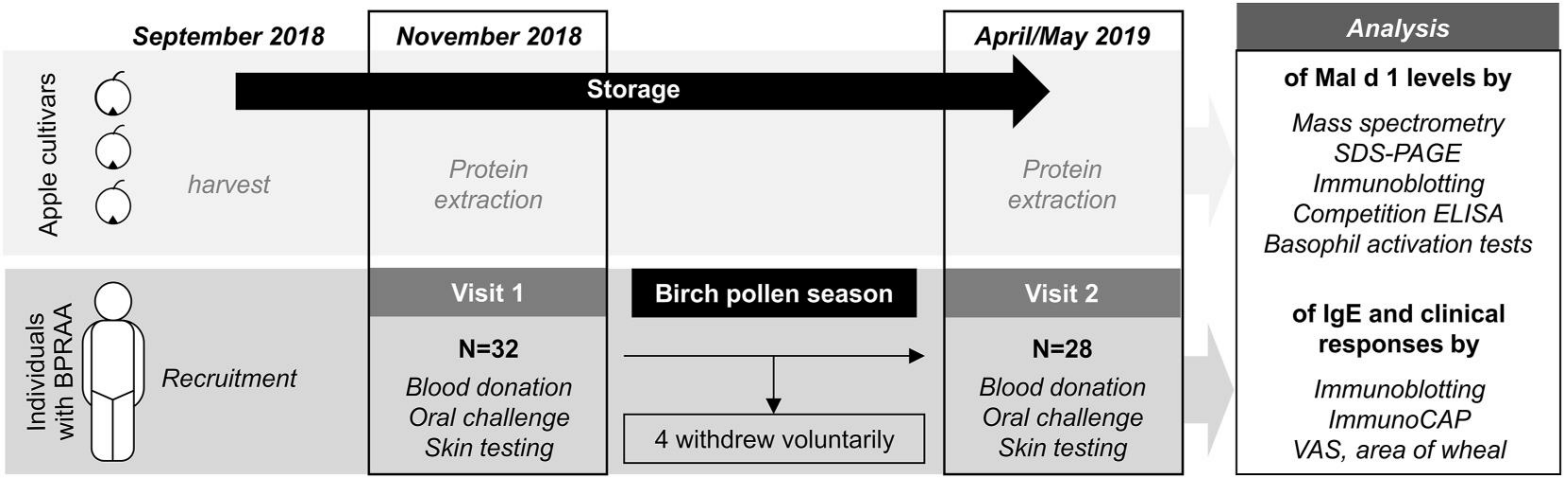
**Study protocol**. Apples were harvested in September 2018. Proteins were extracted after 7 and 29 weeks of storage. Individuals with BPRAA underwent clinical testings with cultivars stored for 7 and 34 weeks, before and after the birch pollen season, respectively. Then, samples and clinical data were analysed, visual analogue score (VAS)

A multidisciplinary approach was set to characterize the allergenicity of three very well storable genuine cultivars with suspected low allergenicity for individuals with BPRAA. The identification and quantification of Mal d 1 by mass spectrometry and immunological assays were complemented with basophil activation tests (BAT) and clinical challenges of allergic individuals. Furthermore, storage conditions known to enhance the concentration of Mal d 1^28^, ^29^, ^30^, ^31^ as well as pollen exposure known to increase the clinical reactivity of allergic individuals were considered.^2^, ^14^ Figure 1 summarizes the longitudinal study protocol.

## 2 MATERIAL AND METHODS

### 2.1 Apple cultivars

The three apple (*Malus* × *domestica* (Borkh.)) cultivars ‘Bay 4069’ (parentage ‘Rafzubin’×‘Pomona’, tradename ‘Gräfin Goldach’), ‘Bay 4152’ and ‘Bay 4210’ (parentage ‘Pinova’×‘Topaz’, tradename ‘Sonnenglanz’) were included. All varieties were bred at Bayerisches Obstzentrum. As references for high or low allergenicity, the varieties ‘Golden Delicious’ (GD) and ‘Santana’ were used. The trees of all apple varieties had been grafted on rootstock ‘M9’ and were trained to a fruit wall system in the experimental orchard block of Bayerisches Obstzentrum at 456 m above sea level in Hallbergmoos, Germany. Varietal identity was determined by pomological determination of each tree in the 4 years preceding the year of harvest. Apple fruits of cultivar ‘Santana’ were harvested on 19.09.2018, of ‘Bay 4210’ on 20.09.2018, of ‘Bay 4152’ on 25.09.2018, of ‘Bay 4069’, and of GD on 26.09.2018, and stored at 1.5°C and a relative humidity of 90%–95% under normal atmosphere. Random samples of the fruits were transferred to the Institute of Pathophysiology and Allergy Research on 07.11.2018 and 10.04.2019 where they were stored in plastic bags at 4°C until further use.

## 3 MASS SPECTROMETRY AND DATA ANALYSES

Details are provided in the online supporting information

## 4 PROTEIN EXTRACTION

Within 5 days after arrival, horizontally cut slices from three fruits of the same cultivar (in total 60 g) were immediately homogenized in 200 ml chilled phosphate buffer (pH = 7.0) supplemented with dithiocarbamate (10 mM), EDTA (0.8 mM), PVPP (4% w/v), PMSF (0.5 mM) and Complete EDTA‐free Protease Inhibitor Cocktail Tablets (Roche Diagnostics, Mannheim, Germany) in a blender. After centrifugation (10,000 g) for 30 min at 1°C, the supernatant was filtered through a folded filter followed by a 0.45 μm filter (Thermo Fisher Scientific, Waltham, Massachusetts, USA) on ice. After dialysis against PBS and overnight (o.n.) volume reduction in a SpeedVac concentrator, the protein concentration was determined by BCA protein assay (Thermo Fisher Scientific). Subsequently, aliquots of each extract were stored at −80°C until further use.

## 5 SDS‐PAGE AND IMMUNOBLOTS

Aliquots of all protein extracts were thawed once and the protein content was measured by BCA (Thermo Fisher Scientific). Proteins (20 μg/lane) were separated by 15% SDS‐PAGE and visualized by Coomassie Brilliant Blue R‐250 (AppliChem, Darmstadt, Germany). Recombinant Mal d 1.0108 (rMal d 1, accession number Q9SYW3, 1 μg/lane) produced in‐house served as positive control.^32^ For immunoblotting, separated proteins were transferred onto a nitrocellulose membrane for 60 min. Mal d 1 was detected by incubation with the murine monoclonal antibody (mAb) BIP1, followed by incubation with a horse‐anti‐mouse‐IgG‐HRP (Cell Signaling Technology, Denvers, Massachusetts, USA) and ECL prime detection reagent (Cytiva, Marlborough, Massachusetts, USA), or a pool of seven sera from the study cohort followed by ^125^I‐goat anti‐human IgE (Demeditec, Kiel, Germany). As loading control, a polyclonal rabbit anti‐binding immunoglobulin protein (BIP2, Agrisera, Vannas, Sweden) was used followed by incubation with a mouse anti‐rabbit‐IgG‐HRP (Jackson Immunoresearch).

## 6 COMPETITIVE ELISA

Microplates (Nunc MaxiSorp, Thermo Fisher Scientific) were coated o. n. with rMal d 1 (1 μg/ml in carbonate buffer, pH = 9.6) at 4°C. After washing twice with PBS/0.05% Tween 20 (PBS‐T), plates were saturated with PBS‐T/1% BSA for 1 h at RT. Then, the mAb BIP1, which had been preincubated with titrated amounts of apple extracts or rMal d 1 as standard for 1 h at RT, was added. Preincubation with an unrelated allergen from cow's milk, Bos d 5, and buffer served as negative controls. After incubation for 60 min at RT, plates were washed 5 times with PBS‐T and bound BIP1 was detected with rabbit anti‐mouse IgG‐HRP (Jackson ImmunoResearch). Amounts of Mal d 1 in apple extracts were quantitatively determined based on the standard curve using rMal d 1.

## 7 BASOPHIL ACTIVATION TESTS (BAT)

Heparinized blood from individuals with BPRAA was incubated with titrated amounts of rMal d 1 or apple extracts in HEPES calcium buffer pH 7.4 containing IL‐3 (2 ng/ml) for 15 min at 37°C. Formyl‐methionyl‐leucyl‐phenylalanine (fMLP, 2 μM, Sigma‐Aldrich) and anti‐IgE (0.5 μg/ml, KPL, Gaithersburg, Maryland, USA) served as positive controls. The reaction was stopped with HEPES/EDTA (20 mM) buffer and cells were stained with anti‐CD123‐PerCP, anti‐CCR3‐APC and anti‐CD63‐PE (all BioLegend, San Diego, California, USA), followed by erythrocyte lysis and flow cytometric analysis on a FACS Canto II (BD Biosciences, San Jose, California, USA). Basophils were defined as CD123^+^CCR3^+^ cells. Basophil activation was expressed as percentage of CD63^+^ cells in reference to unstimulated cells. EC50 values were calculated with the four parameter logistic (4PL) regression model:

$$Y=\text{Bottom}\;+\;X^{\text{HillSlope}}*\left(\text{Top}‐\text{Bottom}\right)/\left(X^{\text{HillSlope}}{+\text{EC}50}^{\text{HillSlope}}\right)$$

## 8 STUDY POPULATION AND CLINICAL TESTS

In total, 32 adult individuals with birch pollen allergy as documented by hayfever in spring, positive skin prick test to birch pollen (ALK‐ABELLO, Hørsholm, Denmark), Bet v 1‐specific IgE levels of >0.35 kU_A_/ml (ImmunoCAP, Thermo Fisher Scientific) and reported OAS to fresh apples were included. Dates for visit 1 were scheduled from November 15‐December 4, 2018, and for visit 2 from April 30‐May 23, 2019. Antihistamines were stopped at least 72 h prior to each visit. Up to six participants were tested simultaneously in a clinical research setting equipped for resuscitation and monitoring of vital signs at the Department of Otorhinolaryngology, Medical University of Vienna, Austria, in accordance with Good Clinical Practice, the Declaration of Helsinki, ethical clearance of the local ethics committee (EK1708/2018), and written informed consent. Four individuals discontinued their participation after completing visit 1 because of lack of time.

Open food challenges and prick‐to‐prick (PtP) testing were performed according to the protocol published by Vlieg‐Boerstra *et al* because it matched our facilities and was of acceptable expenditure of time for the participating volunteers.^19^ Briefly, participants underwent single‐blinded food challenges consisting of one bite from unpeeled apples (approximately 15 g) followed by a piece of 50 g of the same cultivar. After 15 min allergic symptoms were scored by using visual analogue scales (VAS) in the range of 0–100 (0 equal to no symptoms and 100 representing severe symptoms). After waiting until symptoms had disappeared, if needed by intensive flushing of the mouth with water, the procedure was repeated with the next cultivar. Each participant was challenged with all apple cultivars in one session. The order of cultivars was randomized at each testing day. Skin testing was performed on the flexor aspect of the forearms with birch pollen extract (ALK‐ABELLO) and fresh apples. Standardized prick‐test‐lancets (ALK‐ABELLO) were pricked into unpeeled apples and then into the skin. Each cultivar was tested in quadruplicates (two duplicates per arm). The testing positions were randomized at each testing day. Histamine and 0.9% w/v NaCl served as positive and negative control, respectively. The wheal reaction was measured after 20 min and transferred onto a record sheet with transparent adhesive tape. The area of wheals was evaluated using ImageJ (Rasband W.S., National Institutes of Health, Bethesda, Maryland, USA, http://imagej.nih.gov/ij/, 1997–2016). Responses were standardized by dividing the mean wheal area of a cultivar by that obtained for the histamine control.

## 9 ALLERGEN‐SPECIFIC IgE LEVELS

After collection, serum samples were stored at −20°C. Allergen‐specific IgE levels were determined by ImmunoCAP (Thermo Fisher Scientific).

## 10 STATISTICS

Statistical analyses were performed using R version 4.1.1^33^ and IBM SPSS 20.0 software (SPSS, Chicago, Illinois, USA). To assess the effect of season and apple cultivar on different outcome variables mixed models with participant as random effect were fitted. As outcome variables we considered VAS scores, PtP standardized area of wheals, and EC50 values. To account for right‐skewness EC50 values were log transformed, whereas for VAS (which included 0 values) the log(*x*+1) transformation was used. *p*‐values of fixed effects were obtained with the lmerTest package.^34^ Tukey post hoc tests for pairwise comparisons of apple sorts were computed with the multcomp package.^35^ Intra‐class correlation coefficients were computed which illustrate the importance of the random intercept for participant. All tests were two‐tailed and differences were considered significant if *p* ≤ 0.05. Given the exploratory nature of the study we did not consider multiple testing correction for the different endpoints.

## 11 RESULTS

### 11.1 Mal d 1 measurements

**FIGURE 2:**
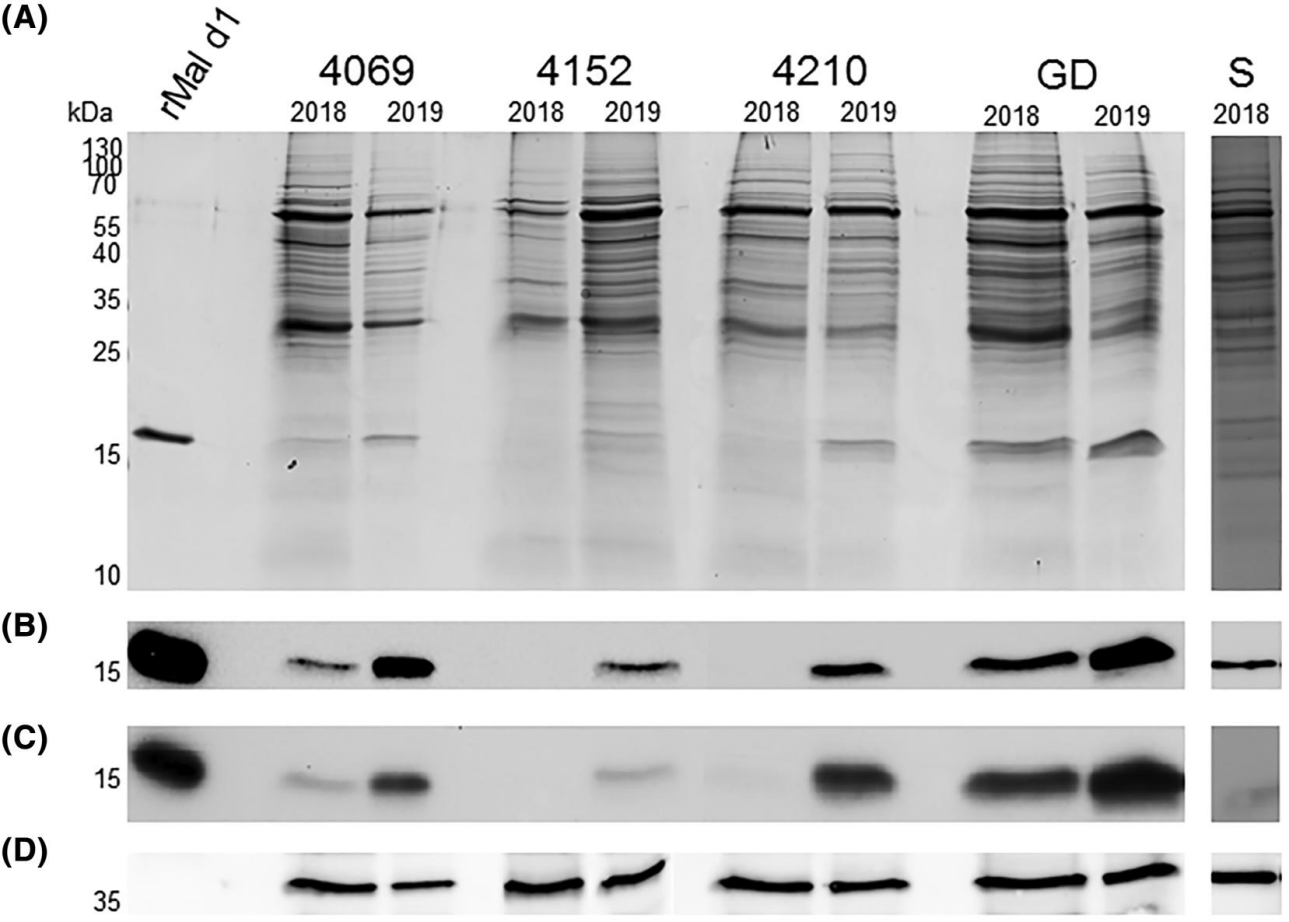
**Detection of Mal d 1 in apple cultivars**. rMal d 1 (1 μg) and protein extracts prepared in November 2018 and April 2019 were separated by SDS‐PAGE and stained with Coomassie (A); after blotting onto nitrocellulose, Mal d 1 was detected with the mAb BIP1 (B) and a serum pool from allergic individuals (C); anti‐BIP2 antibody as loading control (D); kiloDalton (kDa); ‘Golden Delicious’ (GD); ‘Santana’ (S)

As anticipated, ‘Santana’ fruits could only be analysed in November 2018 due to limited storability. The abundance of Mal d 1 in fruits of the genuine cultivars ‘Bay 4069’, ‘Bay 4152’, ‘Bay 4210’, and ‘Golden Delicious’ (GD) in November 2018 and April 2019 was assessed in three ways. First, mass‐spectrometry analysis was used for the identification and quantification of Mal d 1 isoforms described by Pagliarani *et al*.^36^ By comparing equal doses of digested peptides from proteins extracted at both time points an increase of Mal d 1 after storage was detected (Fig. S1). Second, equal concentrations of protein extracts prepared according to Sancho *et al* ^29^ were separated by SDS‐PAGE (Figure 2A) and immunoblots with the mAb BIP1 (Figure 2B) and pooled sera from allergic individuals (Figure 2C) were performed. An anti‐BIP2 mAb served as loading control (Figure 2D). These analyses unanimously revealed enhanced allergen concentrations after long‐term storage in all cultivars and were confirmed by competitive ELISA (Table 1).

**TABLE 1:** Pre‐ and post‐storage Mal d 1 levels (µg/g apple)

	2018	2019
‘Bay 4069’	0.5 ± 0.1	10.4 ± 0.6
‘Bay 4152’	0.03 ± 0.01	0.4 ± 0.1
‘Bay 4210’	0.1 ± 0.04	4.1 ± 0.3
‘Golden delicious’	1.3 ± 0.1	54.4 ± 5.3
‘Santana’	0.3 ± 0.02	‐

## 12 IN VITRO ALLERGENICITY OF APPLE CULTIVARS

**FIGURE 3:**
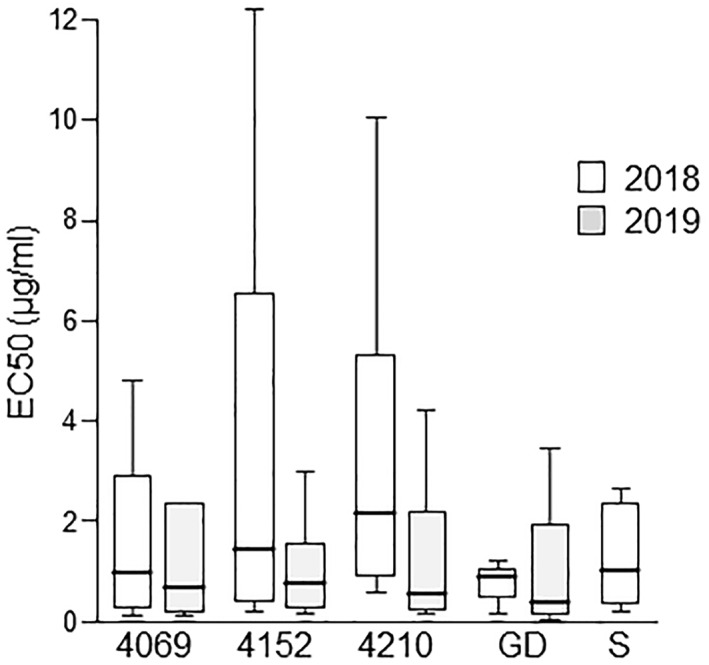
**In vitro allergenicity of apple cultivars.** Basophils from 12 allergic individuals were stimulated with titrated concentrations of apple extracts from November 2018 and April 2019. EC50 values for each individual and cultivar were calculated and summarized in box plots; statistical mixed model analysis is described in the results; ‘Golden Delicious’ (GD); ‘Santana’ (S)

Basophils from 12 allergic individuals were stimulated with equal concentrations of proteins extracted in November 2018 and April 2019. EC50 values, that is, the concentration inducing half‐maximal basophil activation, were calculated for each extract and individual (Figure 3). Mixed model analysis revealed that after long‐term storage EC50 values of all cultivars were significantly decreased (*p* = 0.001), reflecting enhanced allergenicity. Furthermore, the cultivars induced significantly less basophil activation than GD at both time points (*p* < 0.001 for each). ‘Santana’ showed less basophil activation than GD (*p* = 0.069). Moreover, EC50 values of ‘Santana’ and ‘Bay 4069’ were significantly lower than of ‘Bay 4152’ (*p* = 0.013 and *p* = 0.017, respectively). The intra‐class correlation coefficient of 0.8 illustrates that despite a difference in the magnitude of EC50 values between the cultivars their inter‐individual variation was low.

## 13 IN VIVO ALLERGENICITY OF APPLE CULTIVARS BEFORE AND AFTER THE BIRCH POLLEN SEASON

Twenty‐eight individuals with BPRAA underwent single‐blinded open challenges and PtP‐testing with all cultivars in November 2018, that is, before the birch pollen season, and with all cultivars but Santana in May 2019, that is, after the birch pollen season. Their demography is shown in Table 2. Bet v 1‐ and Mal d 1‐specific IgE levels were significantly higher after pollen exposure (*p* = 0.004 and *p* = 0.002, Wilcoxon Signed Ranks Test). Immunoblotting with GD extract revealed that 23 individuals (82%) reacted exclusively to Mal d 1 whereas 5 individuals (18%) showed remarkable IgE‐reactivity to several additional proteins (data not shown). These subjects were excluded from the evaluation of clinical responses as their allergic reactions might be influenced by minor allergens.

**TABLE 2:** Demographics and allergen‐specific IgE levels of study cohort (*n* = 28)

Sex (female/male), *n* (%)	15/13, (54/46)	
Age, median (range)	32 (19–56) years	
	**11/2018**	**5/2019**
**Bet v 1‐specific IgE, median (25%–75% percentile)**	14.5 (6.4–24) kU_A_/l	15.6 (7.2–27.2) kU_A_/l
**Mal d 1‐specific IgE, median (25%–75% percentile)**	4.5 (1.4–8.5) kU_A_/l	5.2 (1.7–10.2) kU_A_/l

**FIGURE 4:**
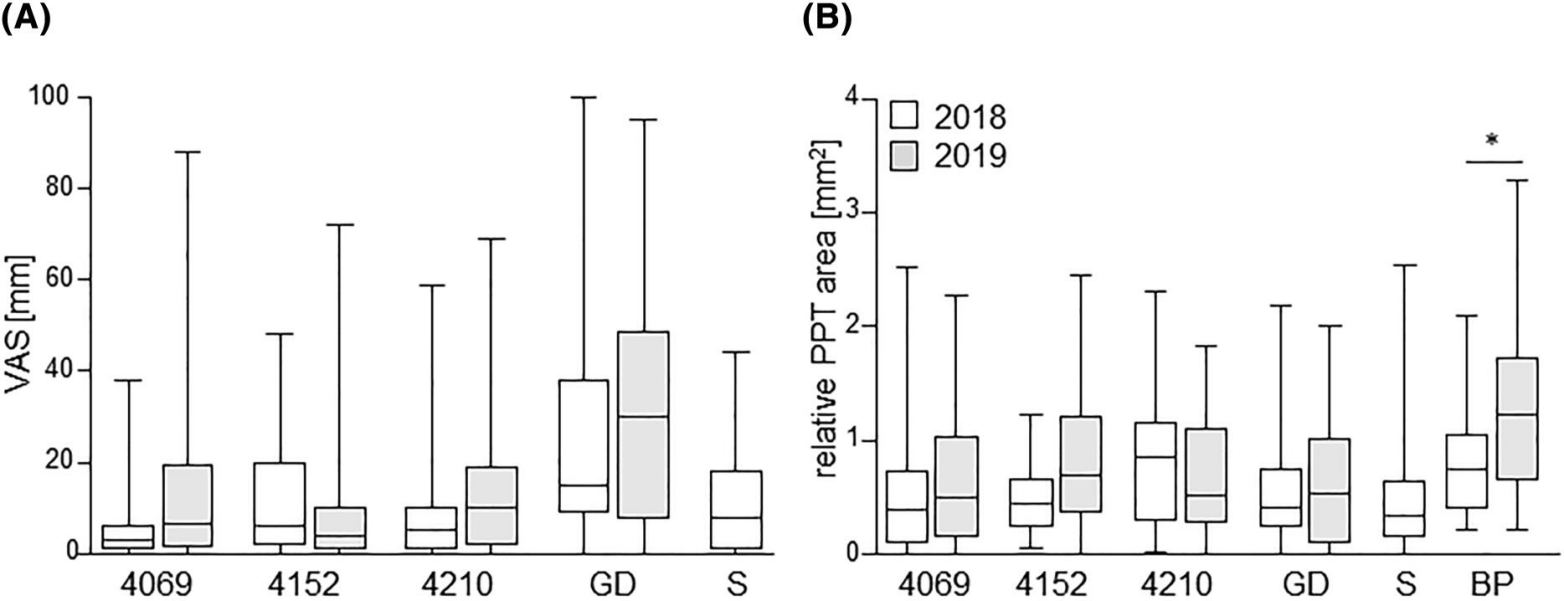
**Clinical reactivity to apple cultivars in November 2018 and May 2019.** Oral symptoms of 23 individuals to 50 g of apple cultivars scored by VAS (A), and prick‐to‐prick‐induced wheal areas normalized to histamine (B); *P < 0.05, Wilcoxon Signed Ranks test; other statistical analyses are described in the results, birch pollen (BP), ‘Golden Delicious’ (GD); ‘Santana’ (S)

VAS in November 2018 showed comparable OAS to ‘Bay 4069’, ‘Bay 4152’, ‘Bay 4210’ and ‘Santana’ (Figure 4A). In May 2019, ‘Bay 4069’, ‘Bay 4210’, and GD induced stronger OAS, however, not statistically different from November 2018 (*p* = 0.21). OAS to ‘Bay 4152’ were comparable at both time points. All cultivars induced significantly less allergic reactions than GD (*p* = 0.007 for ‘Santana’, *p* < 0.001 for all others). The low intra‐class correlation coefficient of 0.14 reflects the strong variability in the subjective reaction to the different cultivars.

Figure 4B displays the results from PtP‐testing. For inter‐patient comparability, all skin reactions were expressed relative to the reactions to histamine which did not significantly differ between both time points (median value 2018 19.6 mm^2^, range 4.2–50.1 mm^2^ and median value 2019 18.1 mm^2^, range 8.1–33.3 mm^2^). There was a clear but insignificant trend of larger PtP reactions after the pollen season and long‐term storage (*p* = 0.079). Moreover, no difference between the different cultivars was found (*p* = 0.34). In comparison, birch pollen‐induced skin reactions increased significantly after the pollen season (*p* = 0.016, Wilcoxon Signed Ranks Test).

## 14 DISCUSSION

Careful monitoring of Mal d 1 levels in the course of storage of four cultivars with an initial concentration of less than 2 μg allergen/g fresh weight has revealed highest concentrations after 28–36 weeks at 4°C without treatment with 1‐MCP.^31^, ^37^ We applied similar conditions to trigger an increase of allergen in the studied cultivars. Untargeted proteomics confirmed a rise of Mal d 1 among all proteins. We also found Mal d 1.02 as the most abundant isoform in the fruits of ‘Bay 4152’, ‘Bay 4210’, and ‘Santana’, matching earlier observations from quantitative PCR.^29^ As this isoform has been described as less allergenic than Mal d 1.01,^38^ we performed competitive ELISA with recombinant Mal d 1.0108 known to induce dose‐dependent skin reactions and OAS in individuals with BPRAA.^39^, ^40^, ^41^ Hereby assessed levels in the fruits of ‘Santana’ were in the range reported by others.^16^, ^24^, ^31^, ^42^, ^43^ The levels in the cultivars in November 2018 were comparable to ‘Santana’ and significantly lower than in GD. BAT confirmed a low initial and storage‐enhanced allergenic potential of the cultivars. Notably, the in vitro allergenicity of ‘Bay 4152’ and ‘Bay 4210’ was significantly lower than ‘Santana’ before storage and comparable thereafter.

The initial low allergenicity of the cultivars was confirmed in open food challenges in November 2018. Often, individuals with BPRAA indicate stronger allergic reactions to apples during and after the birch pollen season,^2^ which has been confirmed in oral challenge tests with GD.^14^ In addition to birch, pollen released by trees containing Bet v 1‐homologous allergens, such as Alder (*Alnus glutinosa*) and hazel (*Corylus avellana*), may trigger Bet v 1‐specific responses through cellular and humoral cross‐reactivity.^44^, ^45^ Indeed, our study cohort displayed significantly higher Bet v 1‐specific IgE levels and skin reactivity to birch pollen after the pollen season which is indicative of a boosted clinical response to the major birch pollen allergen. In parallel, Mal d 1‐specific IgE levels were significantly increased indicating that cross‐reactive IgE antibodies had been boosted. This altered condition was taken into account for the assessment of the cultivars in the second round of food challenges in May 2019. As anticipated, VAS increased, however, remained in the range of ‘Santana’ at visit 1 and significantly lower than GD at both time points. The results from PtP‐testing matched inasmuch as the cultivars ‘Bay 4069’ and ‘Bay 4152’ induced significantly less reactivity than GD in November 2018. In accordance with previous studies, we found no correlation between the size of skin reactions and the intensity of OAS (Fig. S2).^18^, ^19^, ^25^, ^46^, ^47^ Beyond this, the skin reactivity to the cultivars deviated from the results of all other assessments. This discrepancy may be attributed to an unequal distribution of Mal d 1 in the fruits as previously reported for other cultivars.^36^, ^43^ Furthermore, the cultivars may vary in the composition of Mal d 1 isoforms with differing allergenic potential.^24^, ^38^ Accordingly, a detailed isoform‐mapping is currently ongoing. Nevertheless, in accordance with previous studies we conclude that PtP‐testing is no reliable method to characterize the allergenic potential of apples.^19^, ^48^

This multidisciplinary study unified expertise in pomology, plant proteomics, statistics, and allergology to identify apple cultivars with promising characteristics for allergic consumers. Our results show that food challenges with potentially hypoallergenic apples should take place after the birch pollen season to cover both storage‐conditioned allergen levels and the exposure‐conditioned status of allergic individuals. Deduced from the ranking of the allergic potential in open food challenges by mixed model analysis, namely GD>‘Bay 4210’ = ‘Bay4152’ = ‘Bay4069’>‘Santana’, this study introduces three apple cultivars that were tolerated comparably to ‘Santana’. During the open food challenges we noticed that appearance and taste of the fruits are important features that influence the individual evaluation of allergic symptoms. The three apple cultivars deliberately differ in visual nature and flavor, thereby covering individual preferences of consumers. Similar to previous studies the individuals were not asymptomatic after the challenges. Nevertheless, storage with 1‐MCP might further reduce the allergenic potential of the investigated cultivars, as this is a very effective method to inhibit Mal d 1 synthesis.

The calculated ranking of allergenicity by mixed model analysis for BAT was GD>‘Santana’>‘Bay 4069’>‘Bay 4210’>‘Bay 4152’. The discrepancy from the ranking by VAS may result from the use of apple extracts for BAT and of fresh fruits in open food challenges. Moreover, we retrospectively found evidence that our protocol of sequential open challenges with all cultivars on the same day showed a significant trend of milder reactions with every following apple. Still, this result has to be regarded with caution as it is based on a varying number of participants (ranging from 2 to 8) per challenge day and an imbalanced challenge position among the cultivars, for example, ‘Bay 4069’ was never used as second cultivar. The observed trend might have been weakened by more challenge days with an equal number of participants and a selected but not randomized order of the cultivars per day. In view of the independency of BAT from such trends, pollen exposure, and individual preferences and its relative accordance with VAS we recommend BAT with extracts from long‐term stored apples for the screening of cultivars to identify the most promising candidates for food challenges.

## AUTHOR CONTRIBUTIONS

Maria R. Strobl, Verena Ibl, Michael Neumüller, and Barbara Bohle designed the experiments; Maria R. Strobl, Ute Vollmann, Julia Eckl‐Dorna, Astrid Radakovics, Madeleine Schnurer, Martin Brenner, Georgi Dermendjiev, Wolfram Weckwerth, Hilal Demir, and Merima Bublin performed the experiments; Michael Neumüller provided apple cultivars; Maria R. Strobl, Verena Ibl, Florian Frommlet, and Barbara Bohle conceptualization; fundingAcquisition; methodology; projectAdministration; resources; supervision; validation; writing OriginalDraft; writingReviewEditing; Julia Eckl‐Dorna and Christian Müller, resources; Maria R. Strobl, Verena Ibl, Michael Neumüller, Florian Frommlet, and Barbara Bohle wrote the manuscript.

## CONFLICT OF INTEREST

The authors have nothing to declare.

## Supporting information

Supplementary Material

## Data Availability

The data that support the findings of this study are available on request from the corresponding author. The data are not publicly available due to privacy or ethical restrictions.
