# Supplementary material for: Identification of apple cultivars hypoallergenic for birch pollen‐allergic individuals by a multidisciplinary in vitro and in vivo approach
Source: Clin Transl Allergy. 2022 Aug 26;12(8):e12186. doi: 10.1002/clt2.12186 (PMC9412969; doi:10.1002/clt2.12186)
Supplement: Supplementary file 1 — Supplementary Material [file CLT2-12-e12186-s001.docx]

**Online repository**

**Identification of apple cultivars hypoallergenic for birch pollen-allergic individuals by a multidisciplinary *in vitro* and *in vivo* approach**

DI^1^, Ute Vollmann^1^, Julia Eckl-Dorna^2^, MD, Astrid Radakovics^1^, MSc, Verena Ibl^3^, PhD, Madeleine Schnurer^3^, MSc, Martin Brenner^3^, MSc, Georgi Dermendjiev^3^, MSc, Wolfram Weckwerth^3^, PhD, Michael Neumüller^4^, PhD, Florian Frommlet^5^, PhD, Hilal Demir^1^, MSc, Merima Bublin^1^, PhD, Christian Müller^2^, MD, and Barbara Bohle^1^, PhD

^1^Institute of Pathophysiology and Allergy Research, Center for Pathophysiology, Infectiology and Immunology, Medical University of Vienna, Austria

^2^Department of Otorhinolaryngology, Medical University of Vienna, Austria

^3^Department of Functional and Evolutionary Ecology, Division of Molecular Systems Biology, Faculty of Life Sciences, University of Vienna

^4^Bavarian Centre of Pomology and Fruit Breeding, Hallbergmoos, Germany

^5^Center for Medical Statistics, Informatics and Intelligent Systems, Section for Medical Statistics, Medical University of Vienna, Vienna, Austria.

**Mass spectrometry and data analyses**

Within five days after arrival, three fruits of each cultivar were cut horizontally. Slices (1 g) were homogenized in liquid nitrogen using 1 ml of freshly prepared Sucrose SDS-buffer (100 mM Tris-HCl pH 8.0, 30% (w/v) saccharose, 2% (w/v) SDS, 0,5% (v/v), β-mercaptoethanol, 10 mM EDTA, Protease Inhibitor (Roche Diagnostics, Rotkreuz, Switzerland)). After 5 min at room temperature (RT), Roti-phenol (250 µl) was added and samples were vortexed and incubated for 5 min at RT before centrifugation at 20,000×g for 5 min. Supernatants were carefully transferred and the phenol extraction was repeated. Then, proteins in the supernatants were precipitated by mixing with 2.5 volumes of 0.1 M ammonium acetate in methanol. After incubation for 16 h at 20 °C, the samples were centrifuged at 4º C for 5 min at 5000×g. The protein pellets were washed once with 0.1 M ammonium acetate in methanol, and once with ice cold 70% methanol, centrifuged after each washing step at 4º C for 2 min at 18,000xg and dried in a SpeedVac at RT. Subsequently, proteins were solved in urea buffer (8 M urea, 100 mM ammonium bicarbonate, 5 mM dithiothreitol (DTT), Protease Inhibitor) and quantified in a BioRad Bradford Assay with bovine serum albumin (BSA) as standard. Then, 200 μg of total protein per sample were reduced with DTT (5 mM) by shaking with 700 rpm at 37 °C for 45 min. Cysteine residues were alkylated with iodoacetamide (IAA, 55 mM) by shaking with 700 rpm at RT for 60 min in the dark. Alkylation was stopped by increasing the concentration of DTT to 10 mM while samples were shaking at 700 rpm at RT for 15 min in the dark. Thereafter, the urea concentration was diluted to 2 M with 100 mM AmBic/10% acetonitrile (ACN) and CaCl_2_ was added to a final concentration of 2 mM. Trypsin digestion (Poroszyme immobilized trypsin; 5:100 v/w) was performed at 37 °C over night. Peptides were desalted using C18 solid phase extraction columns (Bond Elut SPEC C18, 96 round-well plate, 15 mg, 1 mL, Agilent Technologies, Santa Clara, USA). After solid-phase extraction, the corresponding eluates were dried in a vacuum concentrator. Peptides were resuspended in 2% acetonitrile, 0.1% formic acid (FA) and quantified by a colorimetric peptide assay (Pierce TM Quantitative Colorimetric Peptide Assay, Thermo Fisher Scientific Inc., Waltham, United States). 0.5 µg (0.1 µg/µl) were separated on an ES803, EASY-Spray column, 50 cm x 75 µm ID, PepMap RSLC C18, 2 µm (Thermo Fisher Scientific Inc, Waltham, Massachusetts, United States of America). Peptides were eluted using a 90 min linear gradient from 2 to 40% of mobile phase B (mobile phase A: 0.1% [v/v] FA in water; mobile phase B: 0.1% [v/v] FA in 80% [v/v] ACN) with 300 nl/min flow rate generated with an UltiMate 3000 RSLCnano system. The peptides were measured with a LTQ-Orbitrap Elite (Thermo Fisher Scientific Inc, Waltham, Massachusetts, United States of America) using the following mass analyzer settings: ion transfer capillary temperature 250ºC, full scan range 350-1800 m/z, FTMS resolution 60,000. Each FTMS full scan was followed by up to 20 data-dependent (DDA) CID tandem mass spectra (MS/MS spectra) in the linear triple quadrupole (LTQ) mass analyzer. Dynamic exclusion was enabled using list size 500 m/z values with exclusion width ± 10 ppm for 30 s. Charge state screening was enabled and unassigned and +1 charged ions were excluded from MS/MS acquisitions. Orbitrap online calibration using internal lock mass calibration on m/z 371.101230 was used.

Raw files were processed with MaxQuant v2.0.3.1 ([http://www.maxquant.org](http://www.maxquant.org/)) and blasted against the Mal d 1 sequence according to (1). Peptide identification for each apple cultivar was performed as described (2). Further analyses were performed by Prism9 (<https://www.graphpad.com/series/getting-started/>).

Figure S1. **Label-free quantification (LFQ) of all Mal d 1 isoforms** identified in the apple cultivars in November 2018 and April 2019. Black lines indicate median values of three fruits per cultivar. GD, ‘Golden Delicious’.


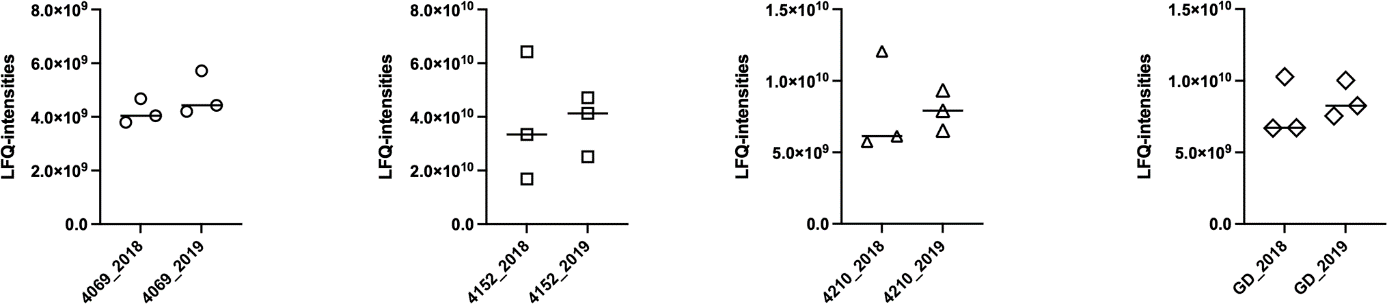


Figure S2. **Correlation of oral challenges and skin testing.** Oral symptoms of 23 individuals scored by VAS were transformed to log(x+1) to account for right-skewness and correlated to PtP-induced wheal areas normalized to histamine


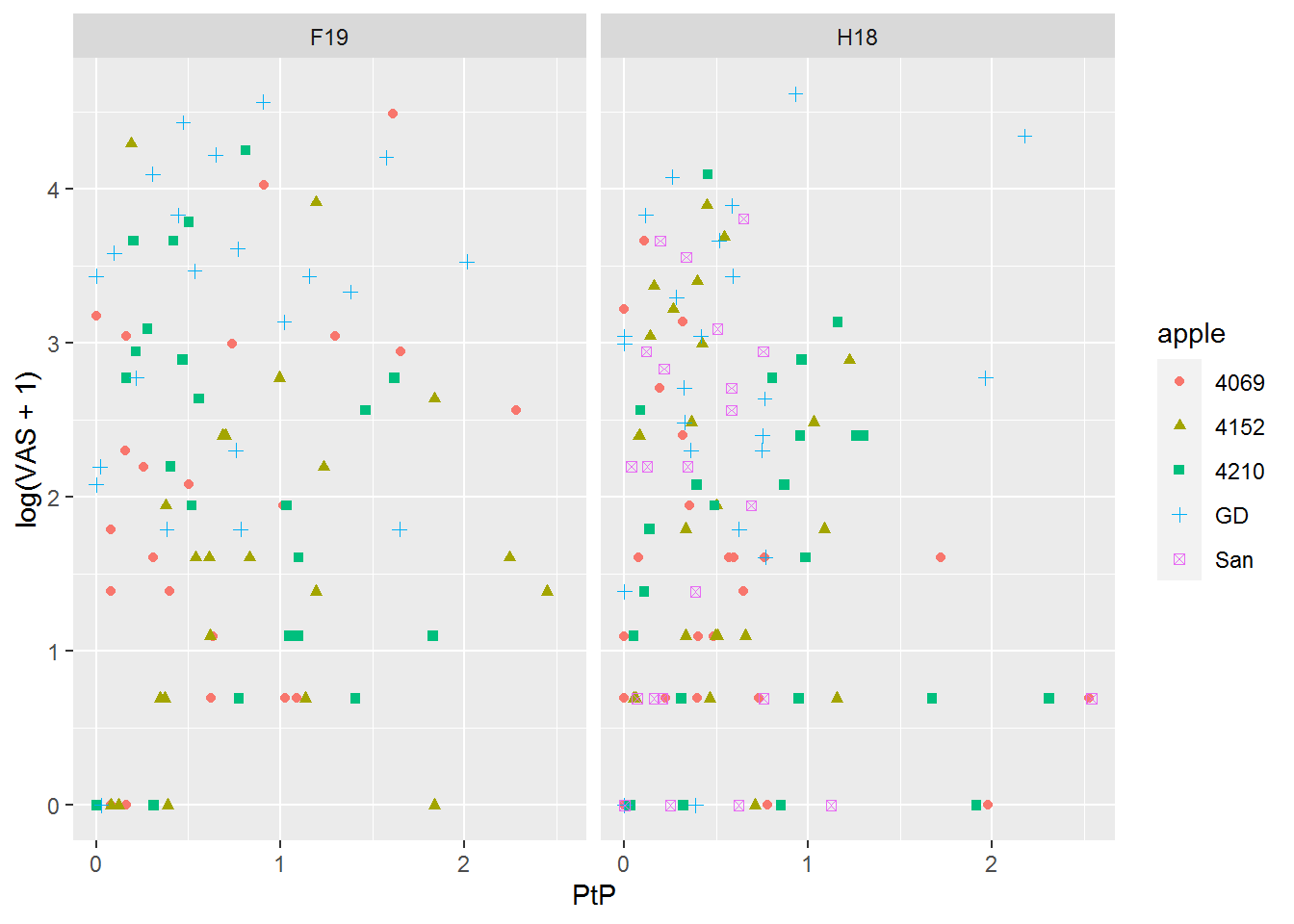


**REFERENCES**

1. Pagliarani G, Paris R, Arens P, Tartarini S, Ricci G, Smulders MM, et al. A qRT-PCR assay for the expression of all Mal d 1 isoallergen genes. *BMC Plant Biol* 2013;**13**:51.

2. Shabrangy A, Roustan V, Reipert S, Weidinger M, Roustan PJ, Stoger E, et al. Using RT-qPCR, Proteomics, and Microscopy to Unravel the Spatio-Temporal Expression and Subcellular Localization of Hordoindolines Across Development in Barley Endosperm. *Frontiers in Plant Science* 2018;**9**.
